# Poster Session II – Poster of Distinction II - A187 FOOD-SPECIFIC CD4^+^ FOXP3^+^ REGULATORY T CELLS IDENTIFY DELAYED-HYPERSENSITIVITY ALLERGIES AND FOOD TRIGGERS IN PEDIATRIC PATIENTS

**DOI:** 10.1093/jcag/gwaf042.187

**Published:** 2026-02-13

**Authors:** C Poloni, A Sze, J Nguyen, E Giles, T Steiner, L Cook

**Affiliations:** The University of British Columbia, Vancouver, BC, Canada; The University of British Columbia, Vancouver, BC, Canada; Department of Paediatrics, Monash Children’s Hospital, Clayton, Victoria, Australia; Department of Paediatrics, Monash Children’s Hospital, Clayton, Victoria, Australia; The University of British Columbia, Vancouver, BC, Canada; University of Melbourne, Melbourne, Victoria, Australia

## Abstract

**Background:**

Eosinophilic oesophagitis (EOE) and food protein-induced enterocolitis syndrome (FPIES) are delayed-hypersensitivity reactions to food proteins with increasing prevalence in Australia and Canada. Currently, there are no blood-based assays to identify EOE/FPIES food triggers, and clinicians rely on endoscopy, food elimination diets, and systemic steroid use to identify and treat disease.

**Aims:**

Here, we present a novel blood-based assay to detect food triggers and identify disease mechanisms in EOE and FPIES paediatric patients.

**Methods:**

24 paediatric patients (12 EOE, 12 non-EOE controls) from Monash Children’s Hospital (Melbourne, Australia) and 8 paediatric patients (8 FPIES) from BC Children’s Hospital (Vancouver, Canada) were enrolled. Peripheral blood was acquired during routine endoscopy. Whole blood was stimulated with food antigens for 44-48 hours. T cell phenotyping was carried out via flow cytometry. Activation was measured based on upregulation of CD25 and OX40, while T cell subsets were identified by surface staining of chemokine receptors and intracellular staining of FOXP3.

**Results:**

There was no significant difference in the frequencies of food-specific CD4^+^ T cells between EOE, FPIES and controls. Strikingly, EOE and FPIES patients had significantly reduced food-specific FOXP3^+^ regulatory T cells (p < 0.0001 and p = 0.003) compared to controls. ROC curves using proportions of food-specific FOXP3^+^ cells within responses to food peptides could discriminate between controls and EOE (AUC: 0.78, p < 0.0001). Importantly, this biomarker could predict FPIES trigger and non-trigger foods (AUC: 0.87, p = 0.0068). Cytokine analysis of whole blood cultures with food antigen revealed significantly elevated levels of IL-6, IFNγ, and TNFα (p = 0.016, p = 0.0081, p = 0.042) in patients compared to controls. These differences were food-specific, as there were no significant differences when stimulating whole blood with childhood vaccine INFANRIX Hexa.

**Conclusions:**

Our blood-based T cell assay can detect immune responses to food peptides. Importantly the FOXP3^+^ proportions of these responses can distinguish EOE/FPIES paediatric patients from controls and predict trigger foods in FPIES patients. Further, EOE/FPIES patients have elevated levels of inflammatory cytokines in culture post food stimulation. Recruitment is ongoing and includes EOE patients with known food triggers to validate predictive capacity and to identify mediators of T cell/oesophageal crosstalk.

**Funding Agencies:**

BC Children’s Hospital Research Institute

